# 1,1′-Biphenyl-2,3,3′,4′-tetra­carboxylic acid monohydrate

**DOI:** 10.1107/S1600536808009689

**Published:** 2008-04-16

**Authors:** Yan Jiang, Jian Men, Chong-Yi Liu, Yan Zhang, Guo-Wei Gao

**Affiliations:** a College of Chemistry, Sichuan University, Chengdu 610064, People’s Republic of China

## Abstract

In the organic molecule of the title compound, C_16_H_10_O_8_·H_2_O, the dihedral angle between the two benzene rings is 42.30 (11)°. Extensive O—H⋯O hydrogen bonding helps to stabilize the crystal structure.

## Related literature

For general background, see: Adadie & Sillion (1991 ▶); Hasegawa *et al.* (1999 ▶); Hergenrother *et al.* (2004 ▶); Iataaki & Yoshimoto (1973 ▶); Yang & Su (2005 ▶). For a related structure, see: Holý *et al.* (2004 ▶).
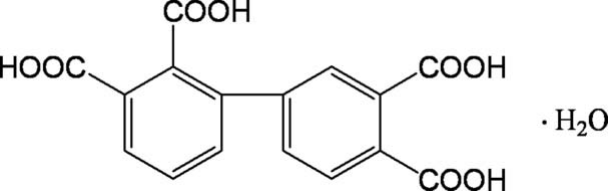


## Experimental

### 

#### Crystal data

C_16_H_10_O_8_·H_2_O
*M* *_r_* = 348.26
Triclinic, 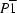

*a* = 6.860 (3) Å
*b* = 11.339 (5) Å
*c* = 11.562 (4) Å
α = 118.14 (3)°
β = 97.34 (3)°
γ = 94.47 (4)°
*V* = 776.7 (5) Å^3^
*Z* = 2
Mo *K*α radiation
μ = 0.13 mm^−1^
*T* = 294 (2) K
0.44 × 0.36 × 0.18 mm

#### Data collection

Enraf–Nonius CAD-4 diffractometer
Absorption correction: none
3389 measured reflections
2889 independent reflections
2074 reflections with *I* > 2σ(*I*)
*R*_int_ = 0.004
3 standard reflections every 250 reflections intensity decay: 1.4%

#### Refinement

*R*[*F* ^2^ > 2σ(*F* ^2^)] = 0.056
*wR*(*F* ^2^) = 0.178
*S* = 0.98
2889 reflections
240 parameters
H atoms treated by a mixture of independent and constrained refinement
Δρ_max_ = 0.35 e Å^−3^
Δρ_min_ = −0.31 e Å^−3^

### 

Data collection: *DIFRAC* (Gabe *et al.*, 1993 ▶); cell refinement: *DIFRAC*; data reduction: *NRCVAX* (Gabe *et al.*, 1989 ▶); program(s) used to solve structure: *SHELXS97* (Sheldrick, 2008 ▶); program(s) used to refine structure: *SHELXL97* (Sheldrick, 2008 ▶); molecular graphics: *ORTEP-3 for Windows* (Farrugia, 1997 ▶); software used to prepare material for publication: *SHELXL97*.

## Supplementary Material

Crystal structure: contains datablocks global, I. DOI: 10.1107/S1600536808009689/xu2413sup1.cif

Structure factors: contains datablocks I. DOI: 10.1107/S1600536808009689/xu2413Isup2.hkl

Additional supplementary materials:  crystallographic information; 3D view; checkCIF report

## Figures and Tables

**Table 1:** Hydrogen-bond geometry (Å, °)

*D*—H⋯*A*	*D*—H	H⋯*A*	*D*⋯*A*	*D*—H⋯*A*
O2—H2⋯O1^i^	0.82	1.87	2.661 (3)	163
O3—H3⋯O6^ii^	0.82	1.89	2.640 (3)	152
O5—H5⋯O9^iii^	0.82	1.76	2.578 (3)	173
O8—H8⋯O7^iv^	0.82	1.84	2.634 (3)	164
O9—H91⋯O4	0.92 (4)	1.84 (4)	2.761 (3)	178 (3)
O9—H92⋯O6	0.86 (5)	1.99 (5)	2.853 (3)	178 (4)

Symmetry codes: (i) 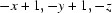; (ii) 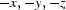; (iii) 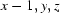; (iv) 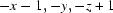.

**Fig. 1.**
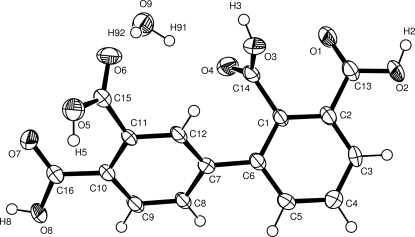
The molecular structure of (I), with displacement ellipsoids drawn at the 30% probability level.

## supplementary crystallographic information

### Comment

Aromatic polyimides are well accepted as high-performance polymeric materials because of their excellent thermal and mechanical properties at elevated temperatures (Adadie & Sillion, 1991); 2,3,3',4'-biphenyltetracarboxylic dianhydride is the most important monomer of aromatic polyimides and particularly useful in the preparation of soluble polyimides with high glass transition temperature and high thermoplasticity (Hasegawa *et al.*, 1999; Hergenrother *et al.*,  2004; Yang & Su, 2005). The title compound is a starting reagent for preparing 2,3,3',4'-biphenyltetracarboxylic dianhydride (Iataaki & Yoshimoto, 1973).

The molecular structure of the title compound is shown in Fig. 1. The dihedral angle between the two phenyl rings of 1,1'-biphenyl-2,3,3',4'-tetracarboxylic acid is 42.30 (11)°, which is markedly differ from 88.69° found in the 1,1'-biphenyl-2,2',3,3'-tetracarboxylic acid monohydrate (Holý *et al.*, 2004). This might be a result of intermolecular O—H···O interactions and steric effects of the title compound. The lattice water molecule links with 1,1'-biphenyl-2,3,3',4'-tetracarboxylic acid via O—H···O hydrogen bonding (Table 1). The extensive O—H···O hydrogen bonding between 1,1'-biphenyl-2,3,3',4'-tetracarboxylic acid molecules helps to stabilize the crystal structure.

### Experimental

2,3,3',4'-Tetramethyl biphenyltetracarboxylate (20.0 g, 52 mmol), concentrated hydrochloric acid (10 ml) and acetic acid (50 ml) in water (50 ml) were refluxed for 4 h. On concentrating the reaction mixture afforded the crude 1,1'-biphenyl-2,3,3',4'-tetracarboxylic acid. Recrystallization of the crude acid from water gave 1,1'-biphenyl-2,3,3',4'-tetracarboxylic acid (m.p. 468–470 K) (Iataaki & Yoshimoto, 1973). Colorless single crystals suitable for X-ray diffraction were obtained at room temperature by slow evaporation of water over a period of several days.

### Refinement

H atoms of the water molecule were located in a difference Fourier map and refined isotropically. Other H atoms were positioned geometrically with C—H = 0.93 Å and O—H = 0.82 Å, and refined using a riding model with *U*_iso_(H) = 1.2*U*_eq_(C,O).

### Crystal data

**Table:**

C_16_H_10_O_8_·H_2_O_1_	*Z* = 2
*M**_r_* = 348.26	*F*_000_ = 360
Triclinic, *P*1	*D*_x_ = 1.489 Mg m^−^^3^
Hall symbol: -P 1	Mo *K*α radiation λ = 0.71073 Å
*a* = 6.860 (3) Å	Cell parameters from 20 reflections
*b* = 11.339 (5) Å	θ = 4.5–7.5º
*c* = 11.562 (4) Å	µ = 0.13 mm^−^^1^
α = 118.14 (3)º	*T* = 294 (2) K
β = 97.34 (3)º	Block, colourless
γ = 94.47 (4)º	0.44 × 0.36 × 0.18 mm
*V* = 776.7 (5) Å^3^	

### Data collection

**Table:**

Enraf–Nonius CAD-4 diffractometer	*R*_int_ = 0.005
Radiation source: fine-focus sealed tube	θ_max_ = 25.5º
Monochromator: graphite	θ_min_ = 2.0º
*T* = 294(2) K	*h* = −8→8
ω/2θ scans	*k* = −5→13
Absorption correction: none	*l* = −13→12
3389 measured reflections	3 standard reflections
2889 independent reflections	every 250 reflections
2074 reflections with *I* > 2σ(*I*)	intensity decay: 1.4%

### Refinement

**Table:**

Refinement on *F*^2^	Secondary atom site location: difference Fourier map
Least-squares matrix: full	Hydrogen site location: mixed
*R*[*F*^2^ > 2σ(*F*^2^)] = 0.056	H atoms treated by a mixture of independent and constrained refinement
*wR*(*F*^2^) = 0.178	Calculated *w* = 1/[σ^2^(*F*_o_^2^) + (0.1334*P*)^2^] where *P* = (*F*_o_^2^ + 2*F*_c_^2^)/3 ?
*S* = 0.98	(Δ/σ)_max_ < 0.001
2889 reflections	Δρ_max_ = 0.35 e Å^−^^3^
240 parameters	Δρ_min_ = −0.31 e Å^−^^3^
Primary atom site location: structure-invariant direct methods	Extinction correction: none

### Special details

**Table:**

Geometry. All e.s.d.'s (except the e.s.d. in the dihedral angle between two l.s. planes) are estimated using the full covariance matrix. The cell e.s.d.'s are taken into account individually in the estimation of e.s.d.'s in distances, angles and torsion angles; correlations between e.s.d.'s in cell parameters are only used when they are defined by crystal symmetry. An approximate (isotropic) treatment of cell e.s.d.'s is used for estimating e.s.d.'s involving l.s. planes.
Refinement. Refinement of *F*^2^ against ALL reflections. The weighted *R*-factor *wR* and goodness of fit *S* are based on *F*^2^, conventional *R*-factors *R* are based on *F*, with *F* set to zero for negative *F*^2^. The threshold expression of *F*^2^ > 2σ(*F*^2^) is used only for calculating *R*-factors(gt) *etc*. and is not relevant to the choice of reflections for refinement. *R*-factors based on *F*^2^ are statistically about twice as large as those based on *F*, and *R*-factors based on ALL data will be even larger.

### Fractional atomic coordinates and isotropic or equivalent isotropic displacement parameters (Å^2^)

**Table:**

	*x*	*y*	*z*	*U*_iso_*/*U*_eq_	
O1	0.3911 (3)	0.4576 (2)	0.0916 (2)	0.0520 (6)	
O2	0.3354 (3)	0.6316 (2)	0.0600 (2)	0.0554 (6)	
H2	0.4355	0.6160	0.0268	0.066*	
O3	0.0748 (3)	0.25686 (17)	0.03658 (17)	0.0392 (5)	
H3	0.1426	0.1961	0.0167	0.047*	
O4	0.2316 (3)	0.32664 (18)	0.24633 (18)	0.0397 (5)	
O5	−0.5242 (3)	−0.05309 (17)	0.10577 (18)	0.0399 (5)	
H5	−0.6058	−0.0072	0.1442	0.048*	
O6	−0.2050 (3)	−0.02764 (18)	0.10600 (19)	0.0467 (5)	
O7	−0.3954 (3)	−0.00002 (18)	0.37645 (19)	0.0455 (5)	
O8	−0.4933 (3)	0.17283 (19)	0.54348 (19)	0.0462 (5)	
H8	−0.5046	0.1186	0.5713	0.055*	
C1	0.0408 (3)	0.4734 (2)	0.2032 (2)	0.0276 (5)	
C2	0.1241 (4)	0.5721 (2)	0.1745 (2)	0.0310 (6)	
C3	0.0495 (4)	0.6933 (3)	0.2149 (3)	0.0387 (6)	
H3A	0.1048	0.7582	0.1958	0.046*	
C4	−0.1069 (5)	0.7174 (3)	0.2834 (3)	0.0481 (7)	
H4	−0.1573	0.7983	0.3103	0.058*	
C5	−0.1882 (4)	0.6206 (3)	0.3119 (3)	0.0385 (6)	
H5A	−0.2929	0.6380	0.3584	0.046*	
C6	−0.1176 (4)	0.4980 (2)	0.2728 (2)	0.0288 (5)	
C7	−0.2085 (3)	0.3987 (2)	0.3089 (2)	0.0267 (5)	
C8	−0.2530 (4)	0.4447 (2)	0.4356 (2)	0.0302 (5)	
H8A	−0.2322	0.5371	0.4954	0.036*	
C9	−0.3284 (4)	0.3537 (2)	0.4735 (2)	0.0309 (5)	
H9	−0.3589	0.3860	0.5583	0.037*	
C10	−0.3590 (3)	0.2150 (2)	0.3867 (2)	0.0273 (5)	
C11	−0.3206 (3)	0.1679 (2)	0.2579 (2)	0.0269 (5)	
C12	−0.2489 (3)	0.2590 (2)	0.2192 (2)	0.0282 (5)	
H12	−0.2273	0.2271	0.1324	0.034*	
C13	0.2963 (4)	0.5479 (3)	0.1050 (3)	0.0346 (6)	
C14	0.1296 (3)	0.3453 (2)	0.1646 (2)	0.0285 (5)	
C15	−0.3477 (4)	0.0196 (2)	0.1528 (2)	0.0301 (5)	
C16	−0.4193 (4)	0.1204 (2)	0.4366 (2)	0.0308 (5)	
O9	0.2047 (4)	0.0732 (2)	0.2263 (2)	0.0527 (6)	
H91	0.211 (5)	0.159 (4)	0.235 (3)	0.066 (10)*	
H92	0.081 (7)	0.041 (4)	0.191 (4)	0.082 (13)*	

### Atomic displacement parameters (Å^2^)

**Table:**

	*U*^11^	*U*^22^	*U*^33^	*U*^12^	*U*^13^	*U*^23^
O1	0.0489 (12)	0.0604 (13)	0.0783 (15)	0.0237 (10)	0.0399 (11)	0.0501 (12)
O2	0.0559 (14)	0.0647 (14)	0.0816 (16)	0.0250 (11)	0.0441 (12)	0.0548 (13)
O3	0.0459 (11)	0.0341 (9)	0.0338 (10)	0.0160 (8)	0.0142 (8)	0.0104 (8)
O4	0.0435 (11)	0.0402 (10)	0.0395 (10)	0.0162 (8)	0.0110 (8)	0.0207 (8)
O5	0.0384 (10)	0.0297 (9)	0.0430 (11)	0.0004 (8)	0.0090 (8)	0.0112 (8)
O6	0.0426 (12)	0.0340 (10)	0.0509 (12)	0.0112 (8)	0.0223 (9)	0.0065 (8)
O7	0.0701 (14)	0.0338 (10)	0.0485 (11)	0.0211 (9)	0.0328 (10)	0.0256 (9)
O8	0.0685 (14)	0.0376 (10)	0.0486 (11)	0.0177 (9)	0.0372 (10)	0.0261 (9)
C1	0.0296 (13)	0.0263 (11)	0.0272 (12)	0.0041 (9)	0.0086 (9)	0.0125 (9)
C2	0.0302 (13)	0.0348 (12)	0.0328 (12)	0.0045 (10)	0.0100 (10)	0.0193 (10)
C3	0.0455 (16)	0.0335 (13)	0.0478 (15)	0.0080 (11)	0.0193 (12)	0.0256 (12)
C4	0.062 (2)	0.0344 (14)	0.0627 (19)	0.0210 (13)	0.0308 (15)	0.0287 (13)
C5	0.0379 (15)	0.0367 (13)	0.0493 (16)	0.0143 (11)	0.0251 (12)	0.0224 (12)
C6	0.0315 (13)	0.0271 (11)	0.0284 (12)	0.0047 (9)	0.0107 (9)	0.0128 (10)
C7	0.0234 (12)	0.0275 (11)	0.0312 (12)	0.0067 (9)	0.0112 (9)	0.0139 (10)
C8	0.0339 (13)	0.0236 (11)	0.0298 (12)	0.0048 (10)	0.0131 (10)	0.0085 (9)
C9	0.0326 (13)	0.0321 (12)	0.0277 (12)	0.0053 (10)	0.0128 (10)	0.0125 (10)
C10	0.0256 (12)	0.0292 (12)	0.0320 (12)	0.0072 (9)	0.0116 (9)	0.0168 (10)
C11	0.0227 (12)	0.0262 (11)	0.0311 (12)	0.0047 (9)	0.0087 (9)	0.0124 (10)
C12	0.0298 (12)	0.0285 (11)	0.0280 (12)	0.0048 (9)	0.0111 (9)	0.0138 (10)
C13	0.0336 (14)	0.0382 (13)	0.0383 (14)	0.0040 (11)	0.0118 (10)	0.0226 (11)
C14	0.0265 (12)	0.0301 (12)	0.0299 (12)	0.0039 (9)	0.0127 (9)	0.0137 (10)
C15	0.0325 (13)	0.0268 (11)	0.0306 (12)	0.0040 (10)	0.0112 (10)	0.0126 (10)
C16	0.0334 (14)	0.0318 (12)	0.0316 (12)	0.0077 (10)	0.0109 (10)	0.0174 (10)
O9	0.0371 (13)	0.0471 (12)	0.0768 (16)	0.0105 (10)	0.0122 (11)	0.0315 (11)

### Geometric parameters (Å, °)

**Table:**

O1—C13	1.214 (3)	C4—C5	1.384 (4)
O2—C13	1.307 (3)	C4—H4	0.9300
O2—H2	0.8200	C5—C6	1.393 (4)
O3—C14	1.318 (3)	C5—H5A	0.9300
O3—H3	0.8200	C6—C7	1.494 (3)
O4—C14	1.213 (3)	C7—C8	1.389 (3)
O5—C15	1.302 (3)	C7—C12	1.403 (3)
O5—H5	0.8200	C8—C9	1.388 (3)
O6—C15	1.218 (3)	C8—H8A	0.9300
O7—C16	1.242 (3)	C9—C10	1.390 (3)
O8—C16	1.286 (3)	C9—H9	0.9300
O8—H8	0.8200	C10—C11	1.394 (3)
C1—C6	1.402 (3)	C10—C16	1.493 (3)
C1—C2	1.409 (3)	C11—C12	1.387 (3)
C1—C14	1.509 (3)	C11—C15	1.518 (3)
C2—C3	1.391 (4)	C12—H12	0.9300
C2—C13	1.483 (4)	O9—H91	0.92 (4)
C3—C4	1.383 (4)	O9—H92	0.86 (5)
C3—H3A	0.9300		
			
C13—O2—H2	109.5	C7—C8—H8A	119.8
C14—O3—H3	109.5	C8—C9—C10	121.0 (2)
C15—O5—H5	109.5	C8—C9—H9	119.5
C16—O8—H8	109.5	C10—C9—H9	119.5
C6—C1—C2	119.7 (2)	C9—C10—C11	119.0 (2)
C6—C1—C14	120.6 (2)	C9—C10—C16	119.1 (2)
C2—C1—C14	119.6 (2)	C11—C10—C16	121.8 (2)
C3—C2—C1	120.2 (2)	C12—C11—C10	119.9 (2)
C3—C2—C13	120.2 (2)	C12—C11—C15	115.4 (2)
C1—C2—C13	119.6 (2)	C10—C11—C15	124.7 (2)
C4—C3—C2	120.0 (2)	C11—C12—C7	121.1 (2)
C4—C3—H3A	120.0	C11—C12—H12	119.4
C2—C3—H3A	120.0	C7—C12—H12	119.4
C3—C4—C5	119.7 (2)	O1—C13—O2	123.5 (3)
C3—C4—H4	120.1	O1—C13—C2	122.4 (2)
C5—C4—H4	120.1	O2—C13—C2	114.1 (2)
C4—C5—C6	121.8 (3)	O4—C14—O3	125.3 (2)
C4—C5—H5A	119.1	O4—C14—C1	122.0 (2)
C6—C5—H5A	119.1	O3—C14—C1	112.5 (2)
C5—C6—C1	118.5 (2)	O6—C15—O5	120.3 (2)
C5—C6—C7	119.4 (2)	O6—C15—C11	119.1 (2)
C1—C6—C7	122.1 (2)	O5—C15—C11	120.3 (2)
C8—C7—C12	118.4 (2)	O7—C16—O8	124.1 (2)
C8—C7—C6	119.5 (2)	O7—C16—C10	120.3 (2)
C12—C7—C6	122.1 (2)	O8—C16—C10	115.5 (2)
C9—C8—C7	120.5 (2)	H91—O9—H92	100 (4)
C9—C8—H8A	119.8		

### Hydrogen-bond geometry (Å, °)

**Table:**

*D*—H···*A*	*D*—H	H···*A*	*D*···*A*	*D*—H···*A*
O2—H2···O1^i^	0.82	1.87	2.661 (3)	163
O3—H3···O6^ii^	0.82	1.89	2.640 (3)	152
O5—H5···O9^iii^	0.82	1.76	2.578 (3)	173
O8—H8···O7^iv^	0.82	1.84	2.634 (3)	164
O9—H91···O4	0.92 (4)	1.84 (4)	2.761 (3)	178 (3)
O9—H92···O6	0.86 (5)	1.99 (5)	2.853 (3)	178 (4)

Symmetry codes: (i) −*x*+1, −*y*+1, −*z*; (ii) −*x*, −*y*, −*z*; (iii) *x*−1, *y*, *z*; (iv) −*x*−1, −*y*, −*z*+1.

## References

[bb1] Adadie, M. J. M. & Sillion, B. (1991). Editors. *Polyimides and Other High-Temperature Polymers* Amsterdam: Elsevier.

[bb2] Farrugia, L. J. (1997). *J. Appl. Cryst.* **30**, 565.

[bb3] Gabe, E. J., Le Page, Y., Charland, J.-P., Lee, F. L. & White, P. S. (1989). *J. Appl. Cryst.* **22**, 384–387.

[bb4] Gabe, E. J., White, P. S. & Enright, G. D. (1993). *Am. Crystallogr. Assoc. Pittsburgh Meet.* Abstract PA104.

[bb5] Hasegawa, M., Sensui, N., Shindo, Y. & Yokota, R. (1999). *Macromolecules*, **32**, 387–396.

[bb6] Hergenrother, P. M., Watson, K. A., Smith, J. G. Jr, Connell, J. W. & Yokota, R. (2004). *Polymer*, **45**, 5441–5449.

[bb7] Holý, P., Sehnal, P., Tichý, M., Závada, J. & Císarová, I. (2004). *Tetrahedron Asymmetry*, **15**, 3805–3810.

[bb8] Iataaki, H. & Yoshimoto, H. (1973). *J. Org. Chem.* **38**, 76–79.

[bb9] Sheldrick, G. M. (2008). *Acta Cryst.* A**64**, 112–122. 10.1107/S0108767307043930 18156677

[bb10] Yang, C.-P. & Su, Y.-Y. (2005). *Polymer*, **46**, 5797–5807.

